# Cancer management challenge in a developing country in COVID-19 pandemic: reflection of a group of Moroccan oncologists

**DOI:** 10.2217/fon-2020-0450

**Published:** 2020-06-25

**Authors:** Hind Mrabti, Narjiss Berrada, Ghislaine Raiss, Hamza Ettahri, Halima Abahssain, Mouna Bourhafour, Souha Sahraoui, Hassan Errihani

**Affiliations:** ^1^Department of Medical Oncology, Institut National d'oncologie, Mohamed V University, 10010 Rabat, Morocco; ^2^Chellah Oncology, 10010 Rabat, Morocco; ^3^Department of Medical Oncology, Regional Center of Oncology, University Hospital, 80000 Agadir, Morocco; ^4^Department of Medical Oncology, Regional Center of Oncology, 32203 Al Hoceima, Morocco; ^5^Centre Mohammed VI Pour Le Traitement Des Cancers, Ibn Rochd University Hospital, 20000 Casablanca, Morocco

**Keywords:** cancer, COVID-19, guidelines, oncology, recommendations, Sars-Cov-2

## Abstract

Management of cancer patients during the COVID-19 pandemic is a worldwide challenge – in particular in developing countries where the risk of saturation of health facilities and intensive care beds must be minimized. The first case of COVID-19 was declared in Morocco on 2 March 2020, after which a panel of Moroccan experts, consisting of medical oncologists from universities and regional and private oncology centers, was promptly assembled to conduct a group reflection on cancer patient's management. The main objective is to protect the immunocompromised population from the risk of COVID-19, while maintaining an adequate management of cancer, which can quickly compromise their prognosis. Recommendations are provided according to each clinical situation: patients undergoing treatment, new cases, hospitalized patients, palliative care and surveillance.

Morocco, like all countries, is fighting in real time against the coronavirus disease 2019 (COVID-19) pandemic. The new assessment of the evolution of the COVID-19 pandemic in the world has caused, to date, 347,381 deaths in 213 countries since its emergence, with 5,542,056 confirmed cases [1]. As soon as the COVID-19 pandemic broke out, the question arose of how to care for our fragile cancer patients in Morocco. Oncologists were faced with a dilemma: on the one hand, the pandemic is killing thousands of people around the world every day; on the other hand, cancer is a deadly disease that causes nearly 10 million deaths annually [2] and the delay or lack of treatment, thereby, is detrimental to those affected. This situation is even more critical in a developing country where it is necessary to rapidly reduce the risk of coronavirus spread and not reach saturation of care structures and resuscitation beds, but also not to lose the major cancer management progress that Morocco has achieved in recent years.

It is clear that delivering cancer care during this crisis is a challenge given the much greater risk of respiratory distress and death in elderly and/or co-morbid patients, particularly those with cancer, compared with the rest of the population [3–5]. This concerns patients with active or past cancer, especially if they have recently received or continue to receive cancer treatment. For new patients, the situation is more serious; the major problem that is likely to arise in this new context is the delay in diagnosis and treatment. This can be dramatic in the case of curable cancers such as lymphomas, malignant germ cell tumors, trophoblastic tumors, etc. The delay in diagnosis may be due to patients delaying their consultation until after the epidemic but also to a very high concentration of caregivers and the healthcare system on COVID-19 at the expense of other pathologies (cancer, myocardial infarction, cerebrovascular accident, etc.) making them collateral victims. A group reflection was conducted on the management of cancer patients in the Moroccan context where the epidemic has not yet reached figures leading to saturation of health structures and where the cancer centers are for the moment exempt from the management of COVID-19. This panel comprised medical oncologists working in different sectors: university oncology centers in Rabat and Casablanca, regional oncology centers in Agadir (south of Morocco) and Al Hoceima (north of Morocco) and representatives of the private sector.

## State-of-play of COVID-19 in Morocco

The first potential risk associated with this pandemic was the repatriation of students in Wuhan, in whom not a single case was reported after the end of their confinement period. Shortly after, the start of the epidemic in Morocco was linked to the declaration of the first case on 2 March 2020 in a patient returning from Italy [6]. Most of the cases recorded in Morocco were imported cases until 30 March 2020 when the discovery of local ‘clusters’ announced the entry into Phase II of the epidemic with more than 80% of cases resulting from local contamination. This phase is defined as the detection of local cases or small outbreaks, the objective during this phase is to limit the virus spread.

**Figure 1.**
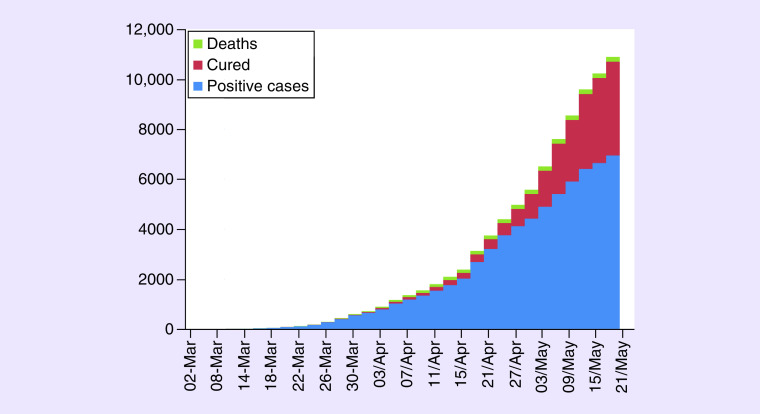
Evolution of the epidemiological situation of COVID-19 in Morocco.

The definition of suspected cases of infection has undergone several updates depending on the epidemiological situation in the country; initially (from 26 February 2020), the possible case was “*anyone with acute respiratory infection with fever and a history of stay in an endemic area*” [7]. As of 24 March 2020 this definition has evolved defining the possible case as “*anyone with an acute respiratory infection who has been in contact with a confirmed case of severe acute respiratory syndrome coronavirus 2 (Sars-Cov-2) infection with or without history of travel*” [8]. Fever was defined, according to the Moroccan ministry of health as temperature higher than or equal to 38°C. To date, Saturday 23 May 2020, the epidemiological situation is characterized by the presence of 7406 confirmed cases, 126,155 negative cases, 4638 patients declared cured and 198 deaths (data updated daily by the Moroccan Ministry of Health, see Figure 1).

Morocco has taken far-reaching and proactive decisions to stem the spread of the virus, ranging from the introduction of border surveillance of arrivals at the various points of entry into the country at the beginning of the crisis, to the total closure of borders and public institutions (schools, cinemas, theatres) imposing general containment of the population and the declaration of a state of health emergency in the country. On 6 April 2020, the government decreed the obligation to wear masks among the population.

In terms of health, the Ministry of Health adopted very early (on 27 January 2020) a plan to monitor and respond to the infection [9], containing a set of measures and organizational recommendations for the management of this crisis. Overall, it is an adaptation of the entire health sector to the evolving epidemiological situation related to COVID-19, with a redeployment of health personnel toward the care of infected patients, the closure of operating rooms and the requisition of resuscitation beds. So far, Moroccan oncology centers have been spared from these measures, although they risk being partially requisitioned if the evolution of the epidemic so requires. This maintains minimal activity, as we will advise in the recommendations below, ensuring that our patients continue their treatment plans while balancing the benefits and risks in these vulnerable patients. Although we think that cancer patients should be kept away from the risk of COVID-19 and that we must avoid admitting COVID-19 patients to oncology facilities, unless all health structures and intensive care units are full.

## International data on COVID-19 in a cancer patient

Data specific to cancer patients available based on recent Chinese and Italian experiences are extremely limited and further studies are needed [4,5,10–13]. The first Chinese study included approximately 1600 patients with laboratory-confirmed acute respiratory illness secondary to COVID-19, 18 of whom had cancer [5].

COVID-19 rate appeared to be higher in cancer patients than in the general population (1 vs 0.29%). In particular, among infected patients, the risk of developing severe respiratory complications requiring intensive care was higher in cancer patients than in noncancer patients (39 vs 8%, p = 0.0003). Old age remained the only risk factor associated with serious events due to SARS-CoV-2 infection in cancer patients. Patients having received chemotherapy or surgery in the months prior to infection had a higher risk of developing severe respiratory complications (OR: 5.34; p = 0.0026). However, these 18 patients represent a heterogeneous group and are not an ideal representation of the entire population of cancer patients [10]. An Italian study reported the characteristics of 355 patients dying from COVID-19: the mean age was 79.5 years, 30% were women, 30% had ischemic heart disease, 35.5% had diabetes and 20.3% had active cancer [11]. More than half of the patients suffered from a serious illness (15 out of 28, 54%) and six had to be admitted to the intensive care unit (21%) [11].

Furthermore, in a separate study from a single institution in Wuhan, China, patients over 60 years of age with non-small-cell lung cancer had a higher incidence of COVID-19 than younger people (4.3 vs 1.8%) [13].

## Organization & recommendations for the care of cancer patients in the Moroccan context 

**Table 1.** Recommendations of cancer treatment during COVID-19 pandemic according to indication and line of treatment.

Situation		Recommendations	Examples
Adjuvant		Continue chemotherapy as programmed	
		Therapeutic de-escalation if possible	Breast cancer • Chemotherapy indicated: six cycles not eight • Luminal A breast cancer: endocrine therapy
			Colon cancer: • Capecitabine alone in the elderly • XELOX, no FOLFOX
Neoadjuvant		Continue chemotherapy beyond the number of scheduled courses of treatment	Ovarian cancer: delay interval debulking surgery beyond three cycles
			Colon cancer with resectable liver metastases: extend neo-adjuvant chemotherapy from 3 to 6 months
Palliative	First line	Continue treatment	
	Second line and beyond	Stop chemotherapy if there is low expected benefit	Lung cancer and pancreatic cancer
	Under treatment and clinical benefit	Continue treatment until progression	
	Stable and well-controlled disease	Therapeutic break	

**Table 2.** Global drug adaptations during COVID-19 pandemic.

Treatment	Drugs	Recommendations
Supportive care measures	G-CSF	Intermediate and high risk of neutropenia
	Erythropoietin	In metastatic disease Hemoglobin <10 g/dl
	Corticosteroids	Reduce using in pre-medication
	Intravenous bisphosphonate injections	Switch to 3 monthly schedule
Chemotherapy	Weekly protocol	Switch to 3 weekly protocol
	Cisplatin	Replace by Carboplatin or oxaliplatin, except malignant germ cell tumors
	5-Fluorouracil	Replace by capecitabine
	Vinorelbine	Use oral presentation
	Metastatic disease	Switch to oral chemotherapy if possible (e.g., breast cancer)
Endocrine therapy	All	No change
Target therapy	All	No change
Immunotherapy	Pembrolizumab	400 mg every 6 weeks
	Atezolizumab	1680 mg every 4 weeks

Several international recommendations have emerged: some recommend continuing to treat cancer patients in the same way [14], while others put forward prioritizing management according to the urgency of the situation or the benefit of the treatment or readjusting therapeutic protocols [15–22].

In Morocco, although we are not yet at a very advanced stage of the epidemic, in the sense that we are not at a saturation of health structures, we believe it is important to put an anticipatory strategy in place. This is, first, to protect our patients and caregivers from overexposure to COVID-19, but also to avoid falling into the trap of excessive fear of COVID-19, which could have an adverse effect on patient management and prognosis.

This fear may result in strategies of eviction from care structures by patients but also in the use of less burdensome and sometimes suboptimal therapeutic strategies on the part of caregivers.

At the start of the pandemic, Moroccan colleagues published a correspondence on how to prepare and reorganize African anticancer centers in the COVID-19 outbreak, they also promoted an anticipatory strategy [23].

The Moroccan expert panel of oncologists, proposed these recommendations for the management of cancer patients which had to take into account several parameters:

- Keep cancer patients away from the risk of contracting the coronavirus: which could be dangerous for them but also for their family since they are immunocompromised and are more likely to contract it and therefore transmit it to those around them;
- Take into account the short-term prognosis of the disease, especially for metastatic diseases;
- Take into account the long-term prognosis for patients treated in a curative situation;
- Protect healthcare workers, families of cancer patients.

We thus find ourselves in several clinical situations:

## Patients undergoing treatment: the general idea is to focus activity & management on patients under treatment

- Categorize patient's management according to the risk benefit for each patient and not by patient group or stage of the disease, as recommended by several guidelines [15,18,19,22];
- Take into consideration the social situation of each patient (distance from home, access to the emergency room, family context, etc.). For patients living outside cities where the cancer centers are located, two problems arise:
  ○ First: travel between Moroccan cities has been blocked, except when traveling with a personal car, and having a special certificate or by ambulance. For these reasons, many patients no longer want to come to their treatments. In these patients, we decided to switch to oral treatment whenever possible to avoid treatment discontinuations;
  ○ Second: the guesthouses where the patients could be accommodated free of charge during the treatment were closed, which meant that the protocol had to be changed to take it over a day (e.g.: Xelox instead of Folfox) or hospitalize the patient (radiotherapy).

In Morocco, the patients are mainly of Muslim religion. In a paper we had previously published, we described how this spiritual side allows them to accept their illness and rely on God [24]. For this reason, it is easier for patients and their families to stop treatment. In other Middle East and North Africa (MENA) regions, things can be different, as illustrated by the report of our Lebanese colleagues [25].

Thus, for patients undergoing systemic cancer treatments, the following is recommended (Table 1 & 2):

- Supportive care measures:
  ○ Use G-CSF as soon as there is an intermediate risk of neutropenia. The rationale behind this recommendation is to avoid immunosuppression, and in particular febrile neutropenia requiring hospitalization. Although there is some controversy about their usage because G-CSF stimulates the granulocytic response and may decrease the lymphocytic response, which is important for the fight against COVID-19 [26];
  ○ For metastatic patients, initiate erythropoietin quickly to reduce the need for transfusions;
  ○ For premedication: significantly reduce the use of corticosteroids, as some experts have suggested the more frequent occurrence of severe forms of COVID 19 in patients on corticosteroid therapy [27];
  ○ Stop intravenous bisphosphonate injections during the entire period of the epidemic were combined with oral therapy alone, by switching to zoledronic acid schedule every 12 instead of 4 weeks [28].
- Replace weekly protocols by 3 weekly regimens;
- Replace cisplatin based-protocols by carboplatin or oxaliplatin according to the indication, except for some curative situations (e.g., malignant germ cell tumors);
- Replace intravenous chemotherapy by oral therapies for more than one cycle (such as oral capecitabine and vinorelbine). In this situation, telemedicine without patient's visit can be used mainly for patients living far from the cancer center, labs controls are also done and sent to the oncologist;
- Continue endocrine therapy and oral molecular-targeted therapies, while avoiding patients coming to the hospital; a telemedecine consultation is preferred;
- Space immunotherapy cycles to reduce clinical visits: using 4-week or 6-week dosing instead of 2- or 3-week;
- In the curative setting (adjuvant and neoadjuvant):
  ○ Continue chemotherapy, by applying the above adjustments;
  ○ Continue neoadjuvant chemotherapy beyond the number of scheduled courses of treatment if access to the operating room is not possible. Examples: in ovarian cancer, delay interval debulking surgery beyond three cycles of chemotherapy, in colon cancer with resectable liver metastases, extend neo-adjuvant chemotherapy from 3 to 6 months.
- For palliative anticancer treatments: act according to age, the patient's general condition, co-morbidities, type of treatment (chemotherapy, immunotherapy, targeted therapy), line of treatment, stage and prognosis:
  ○ Continue chemotherapy in patients with good performance status and benefit from the ongoing treatment, in particular for the first lines of treatment;
  ○ Continue treatment with immunotherapy, when the patient responds well because in theory there is less risk of immunosuppression than with chemotherapy. A cytokine release syndrome, which is contributing to severity of acute respiratory distress syndrome, has been described in patients receiving cancer immunotherapy. But it was described mainly with CAR T-cell therapy, and less frequently with most conventional monoclonal antibodies like anti-PD1 and anti-PDL1, that we use for the treatment of solid tumors [29];
  ○ If the patient is diagnosed with COVID-19, all anticancer treatments, including immunotherapy, must be postponed until total recovery;
  ○ Plan therapeutic break whenever possible, in well-controlled disease;
  ○ Significantly reduce second- and third-line chemotherapy, especially when the expected benefit is low. Examples: lung cancer and pancreatic cancer;
  ○ It is very important to not worsen the prognosis of certain cancers which are likely to respond to treatment, even if they are metastatic. For example: Her2 positive breast cancer, late relapse of some cancers. This situation is the most delicate and each file must be discussed in detail;
  ○ Space out assessments and act according to clinical benefit; in other words, do not prescribe an assessment unless there is evidence of clinical progression.

In all the situations where telemedicine is used, physical consultation is planned in case there's any doubt on any complication for patients under oral treatment, or any sign of disease progression

## New cases: new cases should be taken according to the urgency of the situation & the life-threatening prognosis

- Screening tests must be stopped during the epidemic (mammograms, smears, etc.);
- It is also necessary to learn to be satisfied with a minimalist reference check-up; the simplest check-up is the scanner; the endoscopy trays are mostly closed;
- Treatment should be initiated according to the urgency of the situation and the risk–benefit balance;
- With regard to systemic medical treatments (Tables 1 & 2):
  - Favor oral therapies: capecitabine, vinorelbine per os, endocrine therapy for breast and prostate cancer;
  - In curative situations, opt for therapeutic de-escalation if possible and maintain adjuvant chemotherapies providing a significant benefit. Some of the most frequent examples in curative situations are:
    - For luminal breast cancers, without risk factors, especially in borderline situations: favor endocrine therapy. In triple negative and Her2 positive breast cancers, the standard treatment must be used. It should not be forgotten that the strategy is curative and that patients, after the end of the pandemic, risk relapsing into metastatic situation and their vital prognosis will be at stake;
    - For colon cancer in adjuvant situation: do not use chemotherapy in stage II, which is optional; in stage III: the duration of chemotherapy varies from 3 to 6 months depending on the risk: prefer protocols based on capecitabine alone in the elderly or XELOX in others;
    - Waiting strategies need to be put in place: for example, in prostate cancer, defer radiation therapy; in localized and low-volume metastatic cancers, start endocrine therapy.
  - In a metastatic situation, the prognosis is quickly at stake and treatment should be started as soon as possible. Avoid treating elderly patients with co-morbidities and altered performance status, for whom the expected benefit of systemic therapy is low. Of course, in this situation, oral therapies, spaced cures, outpatient treatment should be favored: replace cisplatin with carboplatin as much as possible, avoid intensified protocols and use G-CSF.

## Hospitalized patients

In normal situations, the medical oncology hospitalization unit is dedicated to patients receiving long chemotherapy protocols or requiring special precautions (hyperhydration, close monitoring, etc.). This unit is also responsible for the care of patients with complications related to the disease or treatment, in addition to patients requiring palliative care.

During the pandemic period, the management of the inpatient unit poses several challenges for healthcare teams: in particular, reducing the number of inpatient beds, reviewing the selection criteria for patients requiring hospitalization, changing the way visits are managed, setting up a suitable organization to support altered patients, as well as measures to protect patients and the healthcare team from the risk of coronavirus infection.

Internationally, some teams have reduced the number of inpatient beds in order to keep only those patients in the unit who are tired and require close monitoring [30]. Others recommend the development of strict triage procedures at admission points to select patients requiring hospitalization from those with oncology emergencies while taking into account the simultaneous assessment of COVID-19 symptoms [18].

A readjustment of patient admission conditions and the adoption of certain precautionary measures after admission are suggested, as follows:

- Inpatient admission conditions: prior to admission, the medical oncologist should consider the admission criteria outlined below and be alert for any symptoms that may be related to COVID-19;
  - Curative situation: maintain admission for patients receiving long protocols or requiring special precautions when administering chemotherapy, including hyperhydration (with cisplatin). Although the majority of protocols containing cisplatin should be switched to carboplatin, with the exception of certain situations where there is a major benefit such as germinal tumors;
  - Palliative situation: maintain the admission of patients with metastatic or locally advanced and symptomatic disease (e.g., disseminated small cell lung carcinomas). For asymptomatic or paucisymptomatic patients, it is recommended, depending on the case, to transfer them to a day hospital or to widen the interval between courses of treatment;
  - Oncological emergency situation: maintain the admission of patients with complications due either to the cancerous disease (spinal compression, intracranial hypertension, hypercalcemia, etc.) or to the toxicity of the chemotherapy received (severe anemia and thrombopenia, febrile neutropenia, grade III and IV mucositis, etc.).
- Precautions against COVID-19 after admission: after admission, certain measures must be put in place to minimize the risk of contamination and at the same time ensure adequate management:
  - Reduce the number of doctors visiting patients in bed;
  - Reduce the number of handover meetings with the nursing team;
  - Accelerate the process of receiving chemotherapy preparations to shorten patient stays;
  - Shorten the length of stay of palliative care patients by providing home care guidelines;
  - Comply with the necessary protective measures against coronavirus for the healthcare team and patients;
  - Prohibit family visits;
  - Reserve accompaniment for exceptional cases of bedridden patients with loss of autonomy;
  - Provide an isolation room, in a separated floor, for patients presenting with symptoms of COVID-19 during their stay in the hospitalization unit. When the positivity is confirmed, we recommend to transfer them in healthcare facilities taking care of COVID-19-positive patients and to keep cancer centers COVID-19 free;
  - Provide a containment procedure for people who have been in contact with a patient with COVID-19 symptoms, during or after discharge from the inpatient unit.

## Palliative care patients

One should also think about palliative care patients and try to keep them at home as much as possible, but maintain contact to adapt treatments or admit them to the hospital if the situation becomes unmanageable at home. A palliative care program exists in Morocco, it essentially provides home consultations with adjustment of oral supportive care treatments, but without the possibility of administrating hospital care (home hospitalization). COVID-19 is an opportunity to use telemedicine in this setting.

## Patients under surveillance

Patients should not be brought in for surveillance consultations; unless they present symptoms of recurrence. In low-risk situations of recurrence, they will be postponed and in high-risk situations telemedicine is preferred.

## Access of patients & their companions to the hospital

In order to rationalize the access of patients and their companions to the hospital, measures must be put in place:

- Encourage all patients and their families to apply barrier gestures, including the wearing of masks, and to respect home confinement outside the days they come to the hospital;
- Provide outdoor triage at the door of each oncology center:
  - Assess the risk of COVID-19 by means of an established questionnaire, with systematic temperature measurement of the patient and accompanying person, if applicable;
  - Prohibit access to care providers, or exceptionally allow only one care provider (loss of autonomy, end of life);
  - Patients with respiratory symptoms and/or fever will undergo a complete risk assessment in an isolation room and will be referred to a COVID-19 management center in suspected cases;
  - Ideally screening should be done from home of all patients coming to the oncology facility. However, in our institution, this precaution is only adopted for patients who will be hospitalized. Patients who are admitted to day hospital or consultation are systematically screened when they arrive at the structure.
- Arrange waiting rooms within the oncology center so that there is an acceptable distance between patients; space out appointments to limit the gathering of people in the same place.

## Research activities

Regarding clinical trials and ongoing projects, deviations may occur. Thus we recommend continuing to monitor previously included patients. The recruitment of new patients should be suspended for several reasons: resource limitations, including doctoral students and administrative staff who were mostly advised to stay at home in order to limit contact with patients, the decrease in oncological activity in general.

## Conclusion

Several strategies have been put in place in Morocco for the fight against cancer, which has made it possible to diagnose the disease at an earlier stage and improve access to cancer care and treatment. The world is at a standstill and in containment, but cancer does not wait. Management on a case-by-case basis and maintaining contact with cancer patients is crucial if we are not to end up with a soaring number of complicated and advanced cancer cases, leading to a new health crisis for cancer patients.

## Future perspective

These recommendations are not set in stone but may change depending on the evolution of the epidemic. It is important to remain cautious during the end of lockdown, which is not yet eased in Morocco, in particular with regard to the maintenance of barrier gestures, the systematic screening of patients for COVID 19 and the triage of patients at the door of the hospital. In the same time we can expect a larger influx of patients and probably at a more advanced stage, it must be not forgotten to recall all the patients whose appointments have been postponed and to plan physical consultation with cancer patients under treatment who have been managed by telemedicine.

**Executive summary**

- Delivering cancer care during coronavirus disease 2019 (COVID-19) crisis is a real challenge given the much greater risk of respiratory distress and death in immunocompromised patients, particularly those with cancer.
- Delayed diagnosis or treatment is detrimental to cancer patients. This delay could be caused by patients' fear of contracting the virus, but also by the concentration of the healthcare system on COVID-19-positive patients.
- A group reflection was conducted on the management of cancer patients in the Moroccan context, comprising medical oncologists from university, regional and private oncology centers.

**State of play situation of COVID-19 in Morocco**

- To date, Saturday 23 May 2020, the epidemiological situation is characterized by the presence of 7406 confirmed cases, 4638 patients declared cured and 198 deaths.
- Several measures were quickly put in place by the Moroccan government to limit the spread of the virus: closing of borders and public institutions, hospitalization of all COVID-19-positive patients, lock-down and finally mandatory wearing of masks.
- Healthcare system has been also reorganized redeployment of health personnel toward the care of infected patients, the closure of operating rooms and the requisition of resuscitation beds. Cancer centers are until now spared from management of COVID-19-positive patients.

**International data on COVID-19 in a cancer patient**

- Data from Chinese and Italian studies suggest more severe forms of COVID-19 in cancer patients, with increased risk of respiratory distress and death.

**Organization & recommendations for the care of cancer patients in the Moroccan context**

**Patients undergoing treatment: the general idea is to focus activity & management on patients under treatment**

- Provide supportive care measures as: G-CSF, erythropoietins for metastatic patients, reduce the use of corticosteroids, switch to zoledronic acid schedule every 12 instead of 4 weeks.
- Protocol adjustments: replace weekly protocols by 3-week regimens, replace cisplatine-based protocols by carboplatin or oxaliplatin (except some curative situations), favor and continue oral regimens: oral chemotherapy, endocrine therapy and oral molecular targeted therapies and space immunotherapy cycles.
- Use telemedicine consultations for patients under oral therapy and plan physical consultation if there is any complication There is the possibility to space Immunotherapy cycles every 4 weeks by keeping the same doses or by increasing the doses: pembrolizumab 400 mg every 6 weeks or atezolizumab 1680 mg every 4 weeks.
- In the curative setting (adjuvant and neoadjuvant): continue chemotherapy, by applying the above adjustments.
- For palliative anticancer treatments: act according to age, the patient's general condition, co-morbidities, type of treatment (chemotherapy, immunotherapy, targeted therapy), line of treatment, stage and prognosis.

**New cases**

- New cases should be taken according to the urgency of the situation and the life-threatening prognosis:
  - Screening tests must be stopped during the epidemic.
  - Use the simplest reference check-up as CT scan.
  - Treatment should be initiated according to the urgency of the situation and the risk–benefit balance.
  - In curative situations, opt for therapeutic de-escalation if possible (like endocrine therapy in breast cancer) and indicate high value-added adjuvant chemotherapies.
  - In a metastatic situation, the prognosis is quickly at stake and treatment should be started as soon as possible. Avoid treating elderly patients with co-morbidities and altered general condition, for whom the expected benefit of systemic therapy is low.

**Hospitalized patients**

- A readjustment of patients’ admission conditions and the adoption of certain precautionary measures after admission are necessary, as follows:
  - Prior to admission, the medical oncologist should be alert for any symptoms that may be related to COVID-19.
  - After admission, certain measures must be put in place to minimize the risk of contamination: reduce the number of doctors visiting patients in bed, comply with the necessary protective measures against coronavirus for the healthcare team and patients, prohibit family visits, provide an isolation room for patients presenting with symptoms of COVID-19 during their stay in the hospitalization unit.

**Palliative care patients**

- Palliative care patients must be kept at home as much as possible, but maintain contact by telemedicine consultation, to adapt treatments or admit them to the hospital if the situation becomes unmanageable at home.

**Patients under surveillance**

- Patients should not be brought in for surveillance consultations; unless they present symptoms of recurrence.
